# IDH1^R132H^ mutation increases radiotherapy efficacy and a 4-gene radiotherapy-related signature of WHO grade 4 gliomas

**DOI:** 10.1038/s41598-023-46335-1

**Published:** 2023-11-11

**Authors:** Xuetao Han, Huandi Zhou, Wei Sun, Liubing Hou, Yanqiang Wang, Hong Wang, Zhongqiang Lv, Xiaoying Xue

**Affiliations:** 1 Department of Radiotherapy, The Second Hospital of Hebei Medical University, No. 215, Heping West Road, Xinhua District, Shijiazhuang, 050000 Hebei China; 2 Department of Central Laboratory, The Second Hospital of Hebei Medical University, Shijiazhuang, 050000 Hebei China; 3 Center of Metabolic Diseases and Cancer Research (CMCR), Hebei Medical University, Shijiazhuang, 050017 Hebei China; 4 Department of Clinical Cancer Prevention, The University of Texas MD Anderson Cancer Center, 1515 Holcombe Blvd, Houston, TX 77030 USA; 5 Department of Neurosurgery, The Second Hospital of Hebei Medical University, No. 215, Heping West Road, Xinhua District, Shijiazhuang, 050000 Hebei China

**Keywords:** Cancer, Oncology

## Abstract

The prognosis for the WHO grade 4 IDH-mutant astrocytoma is better than IDH-wildtype glioblastoma (GBM) patients. The purpose of this study is to explore the potential mechanism of how IDH1 mutation can increase the efficacy of radiotherapy and to establish a risk-score model to predict the efficacy of radiotherapy in WHO grade 4 gliomas. First, we conducted experimental study on the effect of IDH1^R132H^ mutation on glioma cells in vitro. Radiosensitivity of glioma cells was detected by γ-H2AX after 5 Gy radiation. Cell proliferation, migration and invasion were determined respectively by CCK-8, EDU, monolayer cell migration scratch assay and Transwell assay. Then we analyzed IDH1 gene status and the survival of WHO grade 4 glioma patients received radiotherapy in our center and verified our results by analyzing CGGA and TCGA database. For the risk-score model, we use CGGA data to find genetic differences between WHO grade 4 IDH-mutant astrocytoma and IDH-wildtype GBM patients, and determined a 4-gene radiotherapy-related signature through survival analysis by R software. Evaluation and verification through different glioma validation sets and different statistical methods. For in vitro experiments, we established glioma cells stably overexpressing IDH1 wild-type and IDH1-mutant proteins. γ-H2AX assay showed that IDH1-mutant glioma cells had higher radiosensitivity than wild-type. CCK-8 and EDU assay showed that proliferation capacity of IDH1-mutant glioma cells declined. Transwell assay and monolayer cell migration scratch assay also showed that IDH1-mutant glioma cells reduced migration and invasion capabilities. Among the 83 WHO grade 4 glioma patients who received radiotherapy in our center, WHO grade 4 IDH-mutant astrocytoma patients had longer OS and PFS versus IDH-wildtype GBM (P = 0.0336, P = 0.0324, respectively). TCGA and CGGA database analysis had the similar results. Through complex analysis of CGGA and TCGA databases, we established a risk-model that can predict the efficacy of radiotherapy for WHO grade 4 glioma patients. The 4-gene radiotherapy-related signature including ADD3, GRHPR, RHBDL1 and SLC9A9. Patients in the high-risk group had worse OS compared to low-risk group (P = 0.0001). High- and low-risk groups of patients receiving radiotherapy have significant survival differences, while patients who did not receive radiotherapy have no survival difference both in CGGA and TCGA databases. WHO grade 4 IDH-mutant astrocytoma is more radiosensitive than IDH-wildtype GBM patients. Our 4-gene radiotherapy-related signature can predict the radiation efficacy of WHO grade 4 glioma patients, and it may provide some reference for clinical treatment options.

## Introduction

WHO grade 4 glioma is the most common and malignant glioma, with a very poor prognosis^1^. The median overall survival (OS) of WHO grade 4 glioma patients is 15–23 months, and the 5-year survival rate is less than 6%^2^.

The development of genomics in recent years has had a huge impact on the diagnosis and treatment of glioma. Glioblastoma (GBM) stratification based on genomics has profound implications for the diagnosis and treatment of GBM^3^. The 2021 WHO Classification of CNS Tumors divides grade 4 gliomas into WHO grade 4 IDH-mutant astrocytoma and IDH-wildtype GBM^4^. WHO grade 4 IDH-mutant astrocytoma patients have a longer overall survival and better response to therapy than IDH-wildtype GBM patients^5^. This may be due to the differential response of GBM to temozolomide depending on the MGMT status, linked to IDH status^6^. More than 90% of IDH mutations occur at codon 132 of the IDH1 gene, with arginine being replaced by histamine (R132H)^7^. IDH mutation is assumed to be one of the earliest genetic alterations, causing particular oncogenic progression of glioma^8^. IDH1 mutation reduces NADPH production (approximately 38%) and consumes NADPH. Thus, the NADPH pool available to scavenge oxygen radicals is significantly reduced, which ultimately leads to DNA damage and induces apoptosis. This may be a cause of increased radiation sensitivity in glioma^9^. However, the molecular mechanisms that mediate increased survival in glioma patients carrying IDH1 mutations remain unknown.

Radiotherapy is one of the standard treatments for WHO grade 4 gliomas^10^. As early as the nineteenth century, studies have shown that radiotherapy can significantly prolong the survival of high-grade glioma patients, even though the OS of WHO grade 4 glioma patients is still very poor. Studies have shown that the high radioresistance of WHO grade 4 glioma is the main reason for the limited improvement in treatment efficacy^11^. IDH1 mutation is one of the most important molecular features of glioma and is significantly related to the prognosis of patients. However, there are few clinical studies focusing on the relationship between radiotherapy efficacy and IDH1 mutation in WHO grade 4 glioma patients. In this study, we sought to provide information on whether IDH1 gene status is closely related to the efficacy of radiotherapy in WHO grade 4 glioma patients by using statistics from the Chinese Glioma Genome Atlas (CGGA), The Cancer Genome Atlas (TCGA) and our own data. Furthermore, we established IDH1 mutant and wild-type glioma cells and assessed whether they differ in radiosensitivity and malignant biological properties. Finally, we developed an IDH1-related signature for evaluating the prognosis of WHO grade 4 glioma patients with the CGGA dataset.

## Materials and methods

### Cell culture

Human glioma cell lines U87MG and U251MG was obtained from Chinese National Infrastructure of Cell Line Resource and maintained in Dulbecco’s modified Eagle’s medium (DMEM), supplemented with 10% foetal bovine serum (FBS) and 1% penicillin/streptomycin at 37 °C and 5% CO_2_.

### Construction of recombinant expression plasmid

A recombinant pENTER expression plasmid (Vigene Biosciences) containing human IDH1 wild-type (IDH1_wt_) and IDH1^R132^^H^ cDNA was generated. The cDNA was fused in frame with a FLAG tag at the C-terminus by using adapter primers for stable overexpression of IDH1_wt_ and IDH1^R132H^ in glioma cells.

### Screening of stable overexpressing cells

Twenty-four hours after plasmid transfection with Lipofectamine 3000 (ThermoFisher), we screened glioma cells with DMEM containing puromycin (Solarbio). The puromycin concentrations were 2.0 μg/ml for U251MG cells and 1.4 μg/ml for U87MG cells. After 5 days of culture, surviving cells were expanded in normal medium and then western blotting was used to detect the expression of IDH1_wt_ and IDH1^R132H^ proteins, establishing cells stably overexpressing IDH1_wt_ and IDH1^R132H^.

### Western blot analysis

Cells were lysed in RIPA buffer (Beyotime) supplemented with Protein Phosphatase Inhibitor (Solarbio). Equal amounts of total proteins (20 μg) were separated on a 10% SDS–polyacrylamide gel, transferred onto PVDF membranes (Millipore), and blocked at RT with 5% nonfat milk. The PVDF membrane was cropped according to the position of protein marker, and then incubated overnight at 4 °C with primary antibodies (IDH1^R132H^: Dianova, DIA-H09, 1:500; IDH1_wt_: Dianova, DIA-W09, 1:500; FLAG: Proteintech, 1:1000, GAPDH: Proteintech, 1:8000). The following day, the membranes were incubated with HRP-conjugated secondary antibodies and visualized using an enhanced chemiluminescence (ECL) system (BIO-RAD, USA).

### γ-H2AX immunofluorescence staining

DNA double-strand break (DSB) kinetics were measured using γ-H2AX foci immunofluorescence staining. Cells were plated in 8-well plates, incubated at 37 °C for 24 h and treated with 5 Gy irradiation. After washing with PBS, the cells were fixed with 4% paraformaldehyde for 30 min at 0.5 h, 2 h, 6 h, and 24 h after irradiation. The cells were permeabilized with 0.2% Triton X-100 for 20 min and stained for γ-H2AX foci using an anti-γ-H2AX antibody (Cell Signaling Technology, diluted 1:200) for 2 h at room temperature. The cells were then stained using a secondary antibody (Abcam, diluted 1:200) for 1 h at room temperature in the dark. Nuclei were stained with DAPI (Solarbio) for 20 min at room temperature. A fluorescence microscope (Leica Microsystems) was used to acquire images. Finally, ImageJ software was used to count the γ-H2AX fluorescent foci in the nucleus.

### CCK-8 assay for cell proliferation

Following stable transfection, glioma cells were seeded into 96-well plates, and cell viability was measured using the CCK-8 (MCE) assay. A total of 2000–3000 cells were seeded per well for testing, which was performed at the same time point every day for 7 days. At the indicated time points, the cell medium was replaced with medium containing CCK-8 reagent (0.5 mg/mL) and incubated for 2 h. Optical density was measured at 450 nm using a microplate reader (BioTek). Data were collected from triplicate samples in three independent experiments.

### EdU assay

Cells were cultivated in 96-well plates at a density of 8000 cells/well and incubated at 37 °C for 24 h. The cells were cultured with 25 μM EdU (Ribobio) medium diluent for 2 h, fixed for 30 min with 4% paraformaldehyde, and permeabilized with 0.2% Triton X-100 for 20 min. The cells were stained with 1× Apollo^®^ 488 for 30 min, and nuclei were stained with DAPI (Solarbio). The results were observed by fluorescence microscopy (Leica, Wetzlar, Germany), and the proportion of proliferating cells is represented by the ratio of EdU-positive cells to DAPI-positive cells.

### Transwell migration and invasion assays

We performed Transwell assays to measure changes in cell migration and invasion.

Briefly, Transwell plates (8-μm pore size, Corning, USA) were assembled in a 24-well plate, and 2 × 10^4^ cells was seeded into the upper well in 100 μL FBS free culture medium. The bottom compartment contained 600 μL of cell culture medium containing with 10% FBS as a chemoattractant; the cells were incubated for 24 h. After incubation, a cotton swab was used to wipe off nonmigrating cells from the upper surface. The migrated cells on the lower surface were fixed with methanol and stained with crystal violet. The average number of cells per field was determined by counting the number of cells in five random fields per well. For invasion assays, cells were seeded in the upper chamber precoated with Matrigel (Corning, USA) in 100 μl of FBS free medium for 48 h, and cells invading the lower surface were fixed, stained, and visualized, as described above.

### Monolayer cell migration scratch assay

Cells were seeded in six-well plates with cell confluency reaching approximately 80–90%. A scratch was made across the surface with a pipette to form a wound. The cell culture medium was changed to serum-free medium after washing with PBS. The scratch wound was observed using a microscope, and after the initial scratch, images were taken at 0 h, 6 h, 12 h, and 24 h. ImageJ software was used to measure scratch wound distance.

### Total RNA extraction and fluorescence quantitative PCR

Extract total RNA from glioma cells using TRIzol reagent (Invitrogen). Detect the quality and concentration of total RNA in these samples through Nanodrop 2000c. Complementary DNA (cDNA) was synthesized using a revert aid first-strand cDNA synthesis kit (Hifair^®^ III 1st Strand cDNA Synthesis SuperMix for qPCR, YEASEN, Shanghai, China). Quantitative PCR was performed with SYBR Green (Hieff qPCR SYBR Green Master Mix, YEASEN, Shanghai, China) on a qPCR system (Model No. CFX96TM Option Module, Bio-Rad, United States). Apply specific primers for each gene to analyze the expression of these extracted cell samples. Standardize all samples based on the expression of the gene encoding human glyceraldehyde 3-phosphate dehydrogenase (GAPDH) as a reference. The relative expression level is calculated as 2^−[(Ct of target gene) − (Ct of GAPDH)]^. The sequence of primers is listed in Supplementary Table S1.

### Study population

TCGA single nucleotide peptide (SNP) data and corresponding clinical information, including survival information, were downloaded from the database. RNA sequencing data, mRNA microarray data and clinical information were downloaded from the CGGA database. The patient population in this analysis included all WHO grade 4 glioma patients in the CGGA and TCGA databases who received radiotherapy with known IDH1 gene status. The clinical data of glioma patients with WHO grade 4 in TCGA and CGGA database were collected to analyze whether OS and progression-free survival (PFS) were closely related to IDH1 gene status. 282 patients were included in the TCGA database for analysis and 265 patients in CGGA database. In addition, we enrolled 83 WHO grade 4 glioma patients who received radiotherapy in our center from 2017.1 to 2020.10 to detect IDH1 gene status and analyse differences in PFS and OS.

### Genetic differences

CGGA mRNA microarray data were used for gene difference analysis. WHO grade 4 glioma patients were divided into WHO grade 4 IDH-mutant astrocytoma and IDH-wildtype GBM. Differentially expressed genes (DEGs) were analysed using the “limma” package of R software^12^. DEGs, including significantly upregulated and downregulated genes, were screened for subsequent analysis with a false discovery rate (FDR) < 0.05 and absolute log2-fold change (FC) > 0.5.

### Gene signature identification

DEGs were subjected to a univariate Cox regression analysis using the “survival” package of R software. Next, least absolute shrinkage and selection operator (LASSO) analysis^13^ was performed to further sort the prognostic DEG candidates using the “glmnet” package of R software. The regression coefficients of optimal prognostic genes were determined by using a multivariate Cox proportional hazards regression model. The risk score was based on the coefficient of each gene as follows: Risk Score = (exp_gene1_ × coef_gene1_) + (exp_gene2_ × coef_gene2_) + … + (exp_geneN_ × coef_geneN_). Based on the median risk score as the cut-off, WHO grade 4 glioma patients who received radiotherapy were divided into high-risk and low-risk groups, and the survival difference between these two groups was examined by KM survival analysis using the R package “survival”. The effect of risk score and clinical parameters on prognosis, including sex, age, IDH gene status, MGMT gene promoter methylation and chemotherapy, was assessed by univariate Cox proportional hazards regression analysis. Furthermore, multivariate Cox proportional hazards regression analysis was used to determine whether the risk score can be used as an independent prognostic factor in WHO grade 4 glioma patients. Our analysis also included other clinical factors with statistically significant differences (p < 0.05) in univariate Cox proportional hazards regression.

**Figure 1:**
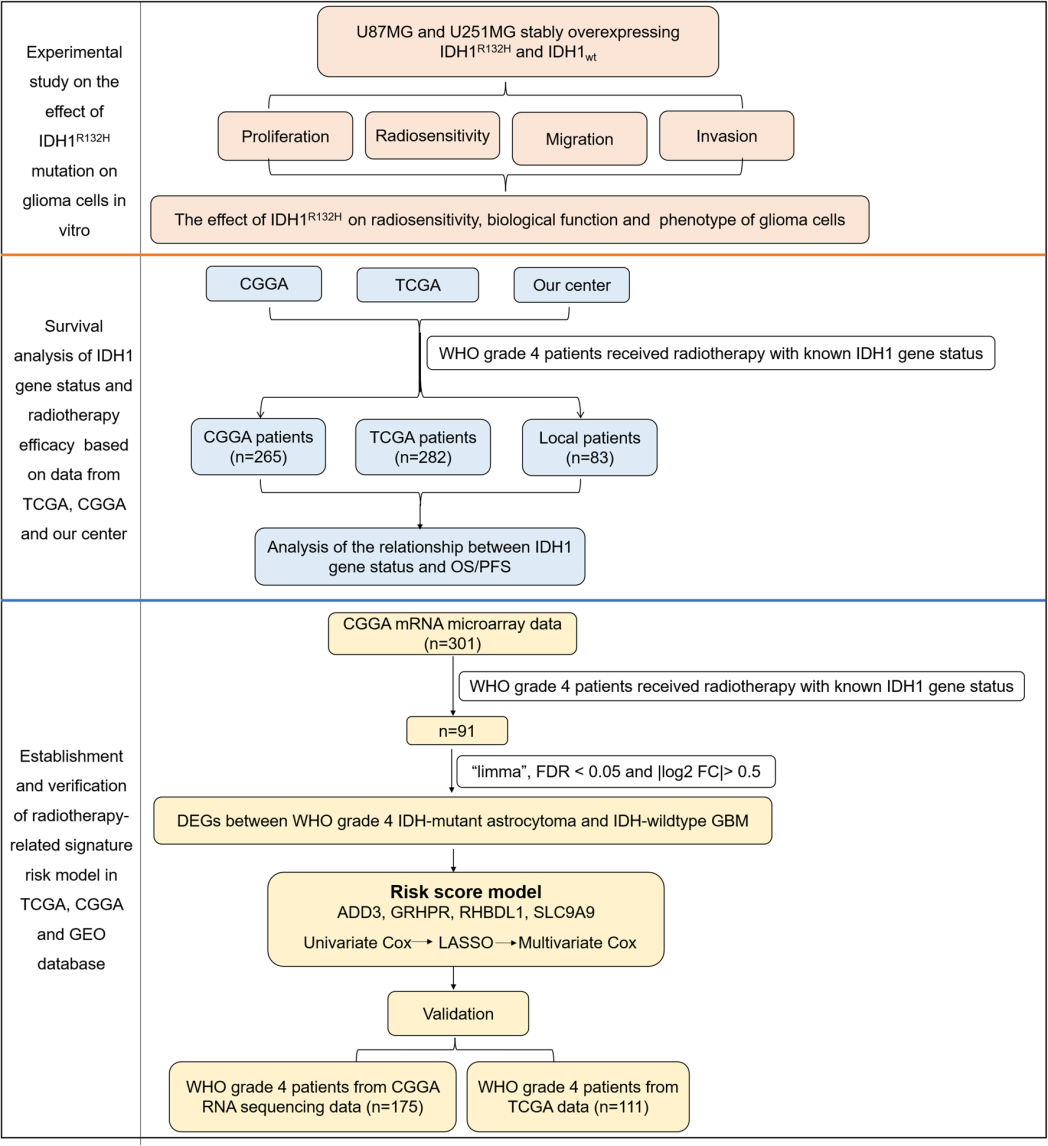
Flowchart for the study. First of all, in vitro, we established two glioma cell lines stably overexpressing IDH1^R132H^ and IDH1_wt_ plasmids. Through the study of radiosensitivity and biological behavior change of the two groups cells, we found that the radiosensitivity of IDH1^R132H^ mutant cells increased and the malignant behavior decreased. Secondly, we enrolled 83 WHO grade 4 glioma patients who received radiotherapy in our center from 2017.1 to 2020.10 to detect IDH1 gene status and analyse differences in PFS and OS. The same patients were enrolled in TCGA and CGGA databases to analyze the correlation between IDH1 gene status and survival. Third, we analyzed the DEGs between IDH1 mutation and IDH1 wild patients in CGGA mRNA microarray data. A 4-gene risk model which can predict the efficacy of radiotherapy was established.

The prediction ability and applicability of the multigene radiotherapy-related signature were verified using CGGA RNA sequencing data. In the validation set, the risk score of each patient was calculated using the coefficients of the genes above. Then, patients were divided into high and low-risk groups based on the medium risk score from the training set. Similarly, KM survival analysis was applied to validate the multigene signature in the validation set. A flowchart of the analysis is presented in Fig. 1.

### Statistical analyses

GraphPad Prism 8 software was used for statistical analysis. Statistical significance was determined using Student’s t test. Data are presented as the mean ± SD, and P < 0.05 was considered statistically significant.

### Ethics statement

This study was conducted to the ethical guideline of the 1975 Declaration of Helsinki. This study has obtained the informed consent of all participants in the manuscript, and was approved by the Ethics Committee of The Second Hospital of Hebei Medical University (No. 2021-P050). No administrative permissions were required to access the raw data.

## Results

### Stable expression of wild-type IDH1 and mutant IDH1^R132H^ constructs in glioma cells

**Figure 2:**
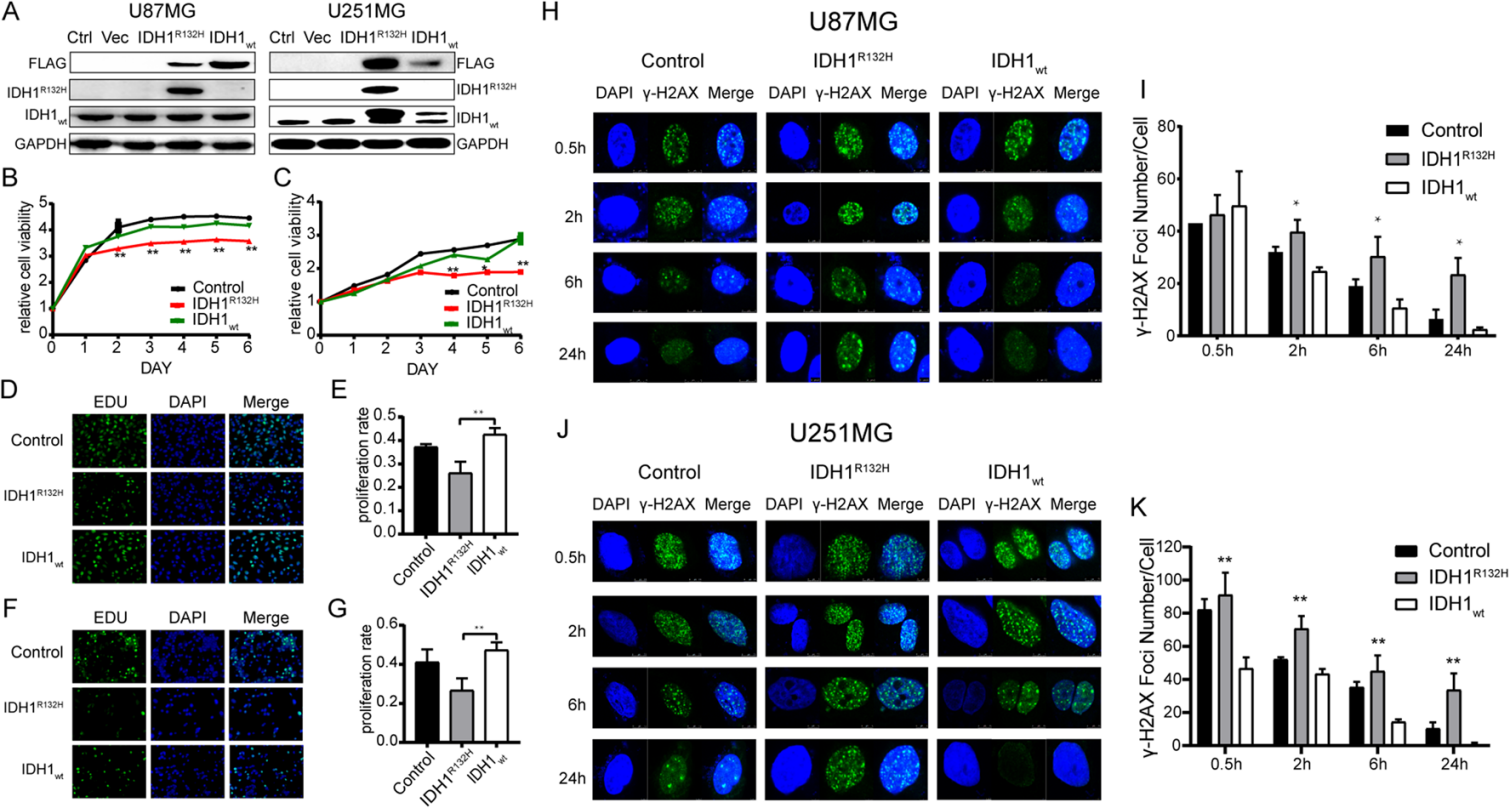
IDH1^R132H^ mutation increased sensitivity of radiation and induced less malignant phenotype in U87MG and U251MG cells. (**A**) Verification after transfection of the wild-type IDH1, IDH1^R132H^ and vector plasmid into U87MG and U251MG glioma cells. (Ctrl: Control, Vec: Vector). (**B,C**) Result of CCK-8 assay in U87MG cell (**B**) and U251MG (**C**) cell. (**D–G**) Result of EDU assay under the microscope (**D**) and corresponding statistics (**E**) of U87MG cell and U251MG (**F,G**). (**H–K**) The formation of γ-H2AX foci at 0.5 h, 2 h, 6 h, 24 h after 5 Gy irradiation in U87MG cell (**H**) and U251MG (**J**) cell and its corresponding statistics (**I,K**).

We conducted in vitro experiments to further study the molecular mechanism of the effects of IDH1 mutation-related radiotherapy. First, we established glioma cell lines that stably expressed IDH1^R132H^ and wild-type IDH1 plasmids. We transfected the wild-type IDH1, IDH1^R132H^ plasmids and empty vector into U87MG and U251MG glioma cells and screened them using puromycin. Western blotting was used to verify stable overexpression (Fig. 2A). The FLAG-tagged IDH1_wt_ and IDH1^R132H^ constructs were stably expressed in U87MG and U251MG glioma cells. The IDH1^R132H^ mutant protein was only expressed in cells overexpressing the IDH1^R132H^ plasmid. Western blot analysis with anti-FLAG, anti-IDH1_wt_ and anti-IDH1^R132H^ antibodies confirmed appropriate expression of IDH1.

### IDH1^R132H^ mutation increases cell sensitivity to radiation

We used immunofluorescence staining to detect the response and radiosensitivity of glioma cells to radiation. H2AX is one of the variants of chromosomal histone protein H2A. When the double stranded DNA of a cell breaks, serine at position 139 on H2AX undergoes phosphorylation modification, forming phosphorylated H2AX (γ-H2AX). The level of γ-H2AX content can clearly reflect the degree of DNA damage and repair. The higher the γ-H2AX content, the more DNA breaks there are. Figure 2H–K shows the formation of γ-H2AX foci in glioma cells at 0.5 h, 2 h, 6 h, 24 h after 5 Gy irradiation. Compared with the control group and IDH1_wt_ group, overexpression of IDH1^R132H^ significantly enhanced radiation-induced γ-H2AX focus formation. Over time, the number of γ-H2AX in the IDH1_wt_ group and control group decreased rapidly, indicating that these cells started DNA repair expeditiously. In contrast, the decrease in the number of γ-H2AX foci in IDH1^R132H^-overexpressing cells was limited, suggesting that DNA damage repair was significantly prevented. Overexpressing IDH1^R132H^ cells exhibited increased sensitivity to radiation compared with the control and IDH1_wt_ groups. These results demonstrate that IDH1^R132H^ mutation affects the response of glioma cells to radiotherapy by regulating DNA damage repair.

### IDH1^R132H^ mutation reduced cell proliferation

We used the CCK-8 assay and EdU assay to measure the proliferation rate of control, IDH1_wt_ and IDH1^R132H^ overexpressing cell lines without irradiation. CCK-8 assay is a widely used kit for evaluating cell survival rate and proliferation ability. The experimental principle of CCK-8 detection is based on changes in cellular mitochondrial respiratory activity, which means that intracellular reducing agents can reduce triazolyltetrazolium in CCK-8 reagents into colored products. This colored product can be quantitatively analyzed through absorbance measurement to evaluate cell count and metabolic activity. As shown in Fig. 2B–G, cells overexpressing IDH1^R132H^ had a significantly lower proliferation rate than the control and IDH1wt groups. EdU is a novel thymidine deoxyribonucleoside analogue that can replace thymidine and be incorporated into newly synthesized DNA during DNA replication. The acetylene group of EdU can covalently react with fluorescent or biotin labeled azides to form a stable triazole ring, thereby detecting cell proliferation through fluorescence detection. The proportion of EdU fluorescence points in IDH1^R132H^-overexpressing cells was significantly reduced compared with that in the other groups. Therefore, both CCK-8 and EdU experiments suggest that IDH1wt groups have stronger proliferative ability.

### IDH1^R132H^ mutation suppresses cell migration and invasion

**Figure 3:**
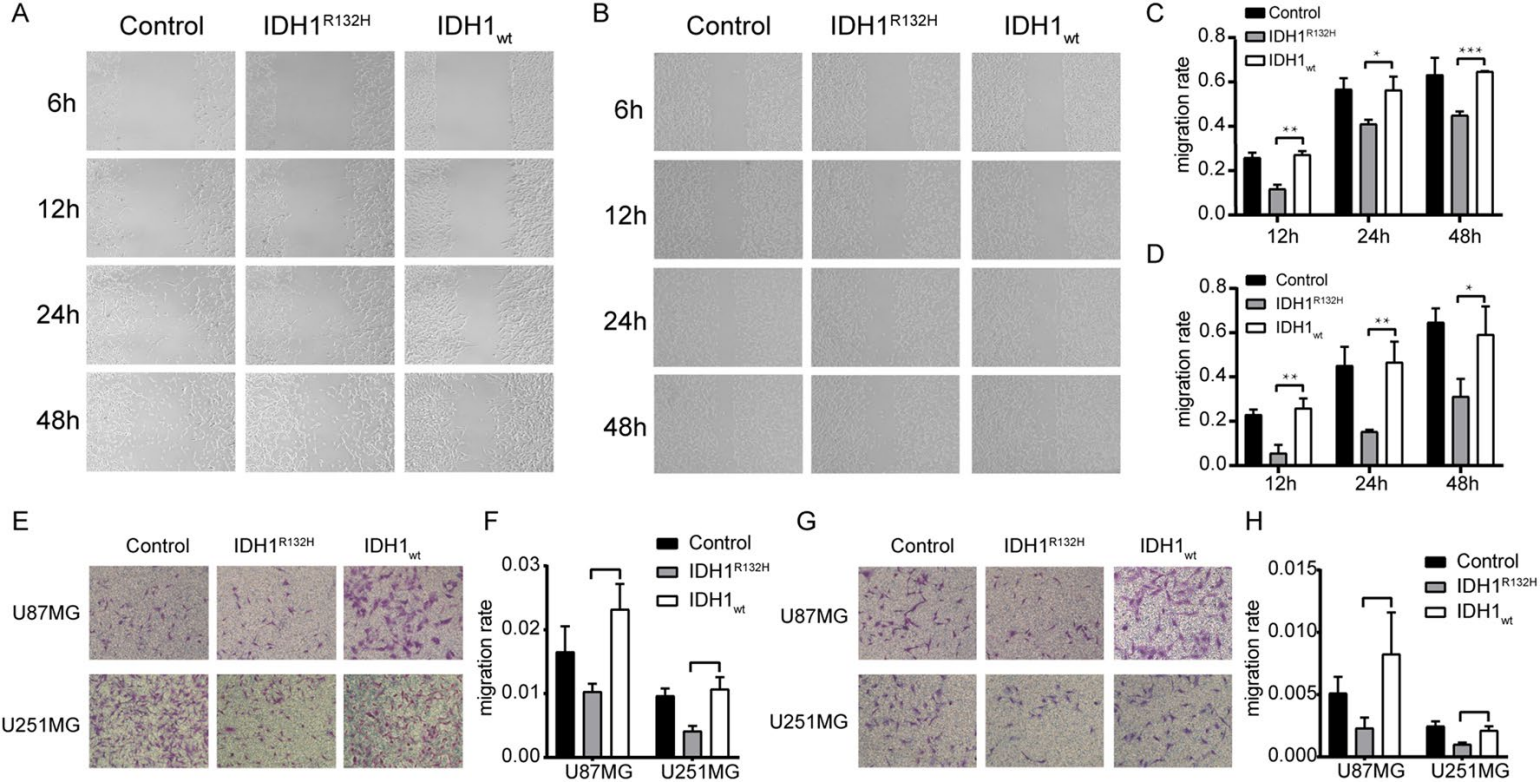
IDH1^R132H^ mutation suppressed migration and invasion in U87MG and U251MG cells. (**A–D**) Result of monolayer cell migration scratch assay and corresponding statistics in U87MG cell (**A,C**) and U251MG cell (**B,D**). (**E,F**) Result of Transwell migration assay under the microscope and corresponding statistics of U87MG cell and U251MG. (**G,H**) Result of Transwell matrigel invasion assay under the microscope and corresponding statistics of U87MG cell and U251MG.

To identify the effects of the IDH1^R132H^ mutation on the migration and invasion of glioma cells, we used Transwell and monolayer cell migration scratch assays. First, we evaluated the rate of cellular migration in control, IDH1_wt_ and IDH1^R132H^-overexpressing U87MG and U251MG cell lines using the Transwell migration assay. As illustrated in Fig. 3, we calculated and compared the number of migrating cells and found that the number in IDH1^R132H^-overexpressing cells was significantly reduced compared to that in IDH1_wt_-overexpressing and control cells. Similar results showing decreased migration of IDH1^R132H^-overexpressing cells were obtained with the monolayer cell migration scratch assay.

We also sought to investigate whether overexpressing the IDH1^R132H^ protein decreases cell aggressivity by using the Transwell invasion assay. As depicted in Fig. 3, we compared the number of invasive cells between the three conditions and detected a significant reduction in the IDH1^R132H^ mutated cells when compared with IDH1_wt_-overexpressing and control cells.

### Survival of WHO grade 4 glioma patients receiving radiotherapy

**Figure 4:**
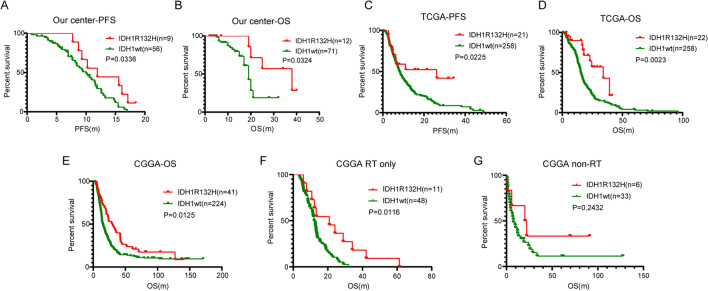
Survival curves of WHO Grade 4 glioma patients with different IDH1 gene status. (**A,B**) Differences in PFS (**A**) and OS (**B**) in WHO Grade 4 glioma patients received radiotherapy in our center from 2017.1 to 2020.10. (**C,D**) Survival analysis of patients in TCGA database. All patients received radiotherapy. The PFS (**C**) and OS (**D**) of WHO grade 4 IDH-mutant astrocytoma patients were better than those of IDH1 wild-type. (**E**) Survival analysis of patients in CGGA database. All patients received radiotherapy. The prognosis of WHO grade 4 IDH-mutant astrocytoma patients was better than that of IDH1 wild-type. (**F,G**) Stratified analysis of patients in CGGA database. Among the patients receiving radiotherapy alone, the prognosis of WHO grade 4 IDH-mutant astrocytoma patients was better (**F**). In the non-radiotherapy group, there was no difference in survival between the two groups (**G**).

We enrolled 83 WHO Grade 4 glioma patients who received radiotherapy in our center from 2017.1 to 2020.10 to detect IDH1 gene status. All patients were assessed for OS, and 65 were assessed for PFS. As shown in Fig. 4A, B, the patients were divided into WHO grade 4 IDH-mutant astrocytoma and IDH-wildtype GBM, and the results showed that the PFS and OS of the WHO grade 4 IDH-mutant astrocytoma were better than IDH-wildtype GBM (P = 0.0336 and P = 0.0324, respectively). Furthermore, we analysed patients in TCGA and CGGA who received radiotherapy and obtained the same results (Fig. 4 C–E). The mPFS of TCGA patients was 26.1 months for the mutation group versus 8.1 months for the wild-type group (P = 0.0225); mOS was 34.1 months versus 15.5 months (P = 0.0023), respectively. Because the CGGA database only contains data regarding the OS of WHO Grade 4 glioma patients, we only analysed the OS of the two groups. Similarly, after radiation therapy, OS was significantly better in the WHO grade 4 IDH-mutant astrocytoma than in the IDH-wildtype GBM, and mOS was 30.6 months versus 15.0 months, respectively (P = 0.0125). A stratified analysis of patients who received radiotherapy alone and those who did not receive radiotherapy was conducted in the CGGA database. In the RT alone group, WHO grade 4 IDH-mutant astrocytoma patients had better survival than IDH wild-type patients (Fig. 4F, P = 0.0116). In patients who did not receive radiotherapy, there was no statistically significant difference in survival between the two groups (Fig. 4G, P = 0.2432), this may be due to the low number of IDH-mutant astrocytoma patients. The lack of stratified analysis of TCGA database patients is due to the small number of WHO grade 4 IDH-mutant astrocytoma patients. The baseline patient characteristics of the TCGA, CGGA and our center cohort are shown in Table 1.

**Table 1:** The baseline patient characteristics of the TCGA, CGGA and our center.

	CGGA (n = 265)		TCGA (n = 282)		Our center (n = 83)	
	Case	Proportion (%)	Case	Proportion (%)	Case	Proportion (%)
Gender						
Male	163	61.5	183	64.9	45	54.2
Female	102	38.5	99	35.1	38	45.8
Age						
≥ 60	63	23.8	136	48.2	24	28.9
< 60	202	76.2	146	51.8	59	71.1
IDH mutation						
Yes	42	15.8	22	7.8	12	14.5
No	223	84.2	260	92.2	71	85.5
Radiotherapy						
Adjuvant	NA	NA	215	76.2	81	97.6
Palliative	NA	NA	42	14.9	2	2.4
NA	–	–	25	8.9	–	–
Chemotherapy						
Yes	202	76.2	231	81.9	83	100
No	62	23.4	33	11.7	0	0
NA	1	0.4	18	6.4	–	–

### Exploration of prognostic DEGs in WHO grade 4 gliomas

**Figure 5:**
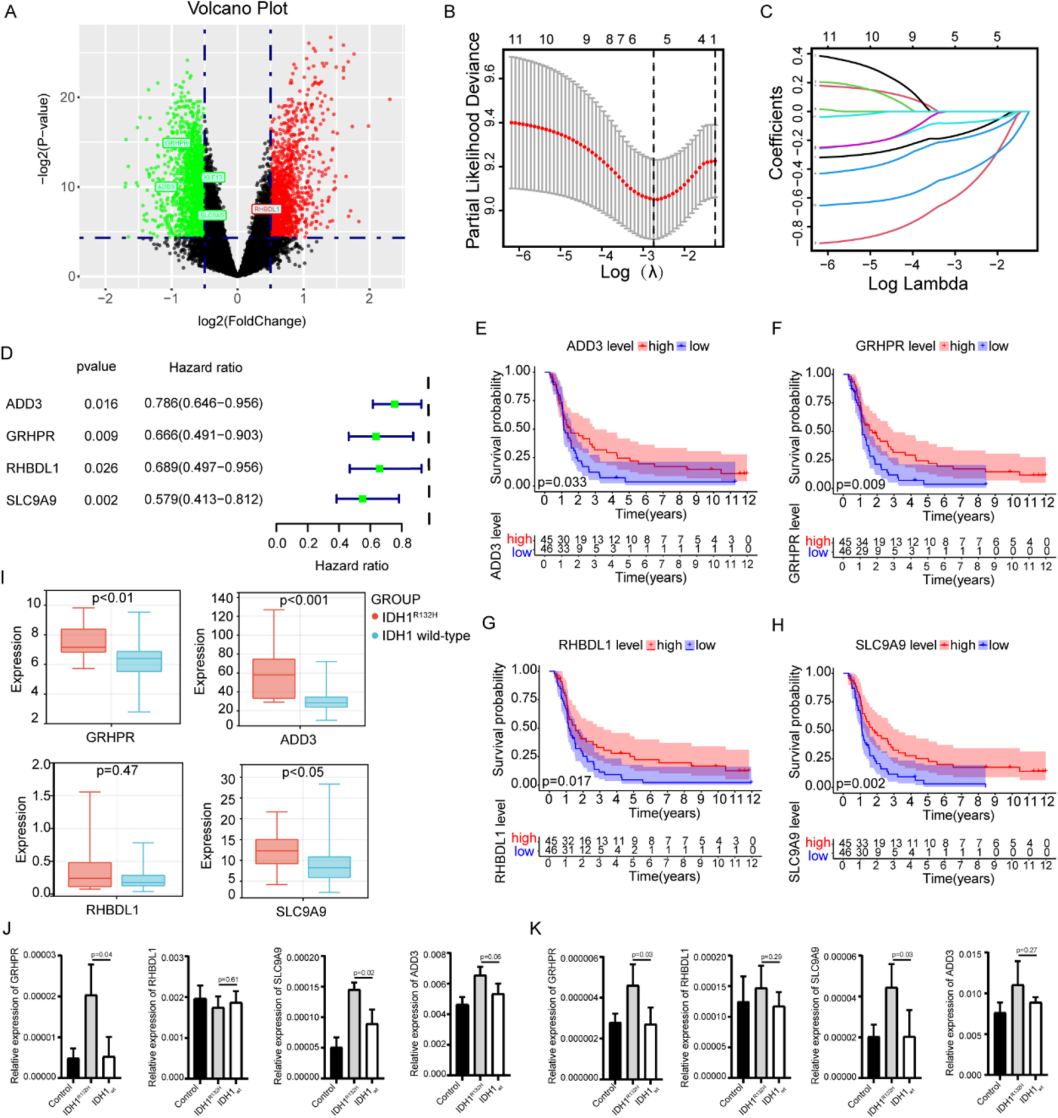
The DEGs between WHO grade 4 IDH-mutant astrocytoma and IDH-wildtype GBM and the establishment of 4-gene signature risk-score model. (**A**) Volcano plot represents the differentially expressed DEGs between WHO grade 4 IDH-mutant astrocytoma and IDH-wildtype GBM, satisfying the criteria of adjusted P-value < 0.05, |log2FC|> 0.5. (**B**) The Cross-validation fit curve calculated by lasso regression method. (**C**) LASSO coefficients profiles of DEGs. (**D**) Multivariate Cox regression analysis of 4 prognostic genes. All 4 genes are protective factors. (**E–H**) The survival curves of high or low expression of ADD3 (**E**), GRHPR (**F**), RHBDL1 (**G**) and SLC9A9 (**H**). The survival time of patients with high expression was significantly better than that of patients with low expression of all 4 genes. (**I**) The expression of ADD3, GRHPR, RHBDL1 and SLC9A9 of WHO grade 4 IDH-mutant astrocytoma and IDH-wildtype GBM patients in GSE121720. IDH mutation group was higher than that in wild group. (**J**) The relative mRNA expression of ADD3, GRHPR, RHBDL1 and SLC9A9 in U87MG glioma cells. (**K**) The relative mRNA expression of ADD3, GRHPR, RHBDL1 and SLC9A9 in U251MG glioma cells.

A total of 91 WHO Grade 4 glioma patients who received radiotherapy were enrolled to identify DEGs in CGGA mRNA microarray data. Of 19,416 genes in the CGGA mRNA microarray dataset, 2237 genes met the criteria (Fig. 5A, P < 0.05, |log_2_FC|> 0.5) after Benjamini & Hochberg procedure. Among them, 1256 were downregulated and 981 upregulated. Using the single variable Cox regression method (set P < 0.001), these 2237 DEGs were used to study the prognostic significance. Initially, 11 prognostic-associated candidate DEGs were identified (Supplementary Table S2). Then, through LASSO regression, 5 genes (ADD3, GRHPR, KLF13, RHBDL1 and SLC9A9) closely related to the prognosis of WHO Grade 4 glioma patients were selected (Fig. 5B, C). Furthermore, multivariate Cox regression was used to establish a radiotherapy-related risk-score model. Finally, 4 DEGs were selected to construct the risk model, including ADD3, GRHPR, RHBDL1 and SLC9A9 (Fig. 5D). The coefficient values and p-values for the 4-gene risk-score model was provided in Table 2. The risk score for each patient was calculated based on the coefficients: Risk score = (−0.25874*Exp ADD3) + (−0.29473*Exp GRHPR) + (−0.80813*Exp RHBDL1) + (−0.55268*Exp SLC9A9). The survival analysis of 4 genes showed that the survival of patients in high expression group was better than that in low expression group respectively (Fig. 5E–H). In order to further verify whether the expression of the 4 DEGs is consistent with the CGGA database, GSE121720 was analyzed. The analysis of GSE121720 dataset also showed that the gene expression in IDH mutation group was higher than that in wild group (Fig. 5I). Furthermore, in order to demonstrate the relationship between the 4 DEGs and the status of IDH1 gene, the relative expression mRNA level of them were identified in glioma cells stably overexpressing IDH1^R132H^ and IDH1_wt_ groups (Fig. 5J, K). The results showed that the expression of ADD3, GRHPR, and SLC9A9 in the IDH1^R132H^ group cells was higher than that in the IDH1_wt_ group, while there was no statistically significant difference in the expression of RHBDL1 between the two groups.

**Table 2:** The coefficient values for the 4-gene risk-score model.

Gene ID	Coef value	HR	HR.95L	HR.95H	P value
ADD3	−0.25874	0.77202	0.58032	1.02705	0.07562
GRHPR	−0.29473	0.74473	0.50363	1.10126	0.13975
RHBDL1	−0.80813	0.44569	0.30160	0.65861	0.00005
SLC9A9	−0.55268	0.57541	0.37904	0.87352	0.00946

**Figure 6:**
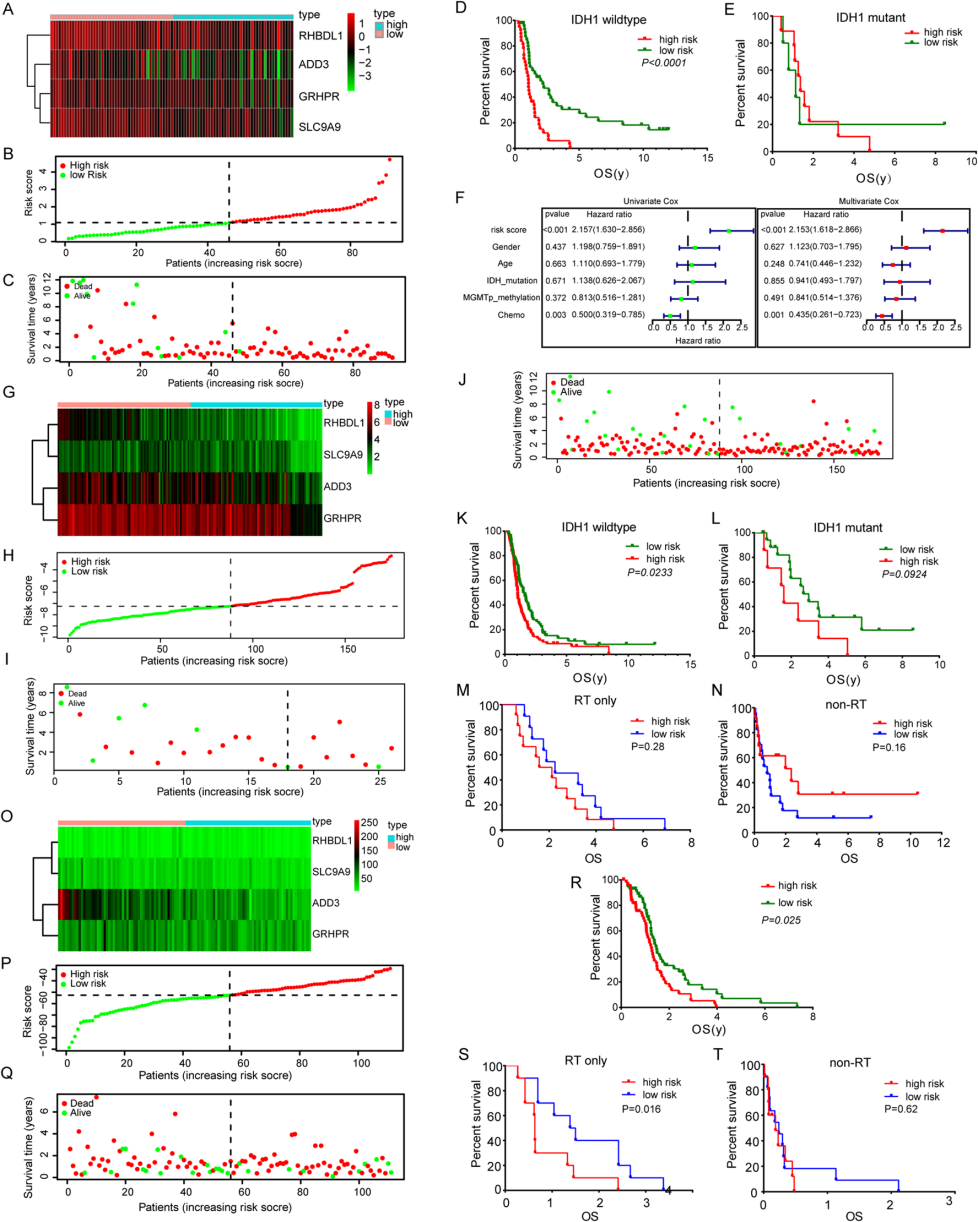
Risk score analysis of 4-gene prognostic model in CGGA mRNA microarray data (**A–F**), CGGA RNA sequencing data (**G–N**) and TCGA data (**O–T**). (**A**) Heat maps of 4 genes in CGGA mRNA microarray data. Rows represent prognostic genes, columns represent samples which is ordered by the assigned risk scores. The expression of 4 genes in high-risk group is low, while that in low-risk group is high. (**B,C**) The curve of risk score (**B**) and survival status (**C**) of the patients. Take the median risk-score as the cut-off value. More dead patients corresponding to the higher risk score. (**D,E**) Kaplan–Meier survival analysis of the 4-gene signature in CGGA mRNA microarray data. Survival between high and low risk group in IDH-wildtype GBM (**D**) and WHO grade 4 IDH-mutant astrocytoma (**E**). In IDH-wildtype GBM patients, the survival rate of the high-risk group was significantly lower than that of the low-risk group. While for WHO grade 4 IDH-mutant astrocytoma, there was no difference in survival between the two groups, which may be due to the small number of cases (only 14 cases). (**F**) Univariate and multivariate Cox regression analysis of 4-gene signature risk-score. The risk-score is an independent prognostic risk factor. (**G**) Heat maps of 4 genes in CGGA RNA sequencing data. Rows represent prognostic genes, columns represent samples. The higher the gene expression, the lower the risk score. (**H**) The curve of risk-score of the patients in CGGA RNA sequencing data. Take the median risk-score as the cut-off value. (**I,J**) Survival status in WHO grade 4 IDH-mutant astrocytoma (**I**) and WHO grade 4 gliomas (**J**). Most of the WHO grade 4 IDH-mutant astrocytoma patients were assigned to the low-risk group. (**K,L**) Kaplan–Meier survival analysis of WHO grade 4 glioma patients treated with radiotherapy in in CGGA RNA sequencing data. Survival between high and low risk group in IDH-wildtype GBM (**K**) and WHO grade 4 IDH-mutant astrocytoma (**L**). (**M**) Survival of WHO grade 4 glioma patients treated with radiotherapy alone in CGGA data. (**N**) Survival of WHO grade 4 glioma patients treated without radiotherapy in CGGA data. (**O**) Heat maps of 4 genes in TCGA data. Rows represent prognostic genes, columns represent samples. (**P,Q**) The curve of risk score (**P**) and survival status (**Q**) of the patients in TCGA data. Take the median risk-score as the cut-off value. The survival time of patients in the low-risk group is longer. (**R**) Kaplan–Meier survival analysis of WHO grade 4 glioma patients in TCGA data. The prognosis of patients in low-risk group is better than that in high-risk group. (**S**) Survival of WHO grade 4 glioma patients treated with radiotherapy alone in TCGA data. (**T**) Survival of WHO grade 4 glioma patients treated without radiotherapy in TCGA data.

WHO Grade 4 glioma patients were divided into a low-risk group and a high-risk group based on the median risk score as the cut-off point (Fig. 6A–C). We analyzed the survival of high- and low-risk groups in WHO grade 4 IDH-mutant astrocytoma and IDH-wildtype GBM respectively. The results showed that there was a survival difference between the two groups in IDH-wildtype GBM (P < 0.0001), but not in WHO grade 4 IDH-mutant astrocytoma (Fig. 6D, E). This may be due to the fact that there are only 14 cases of mutant patients. Univariate Cox regression analysis showed a statistical correlation between 4-gene signature risk-score and OS in CGGA patients (HR = 2.157, P < 0.001, Fig. 6F). After adjusting for other confounding factors, the risk-score remains an independent prognostic indicator for OS (HR = 2.153, P < 0.001, Fig. 6F).

### Verification of the 4-gene signature risk model

To validate the predictive capability of the 4-gene prognostic signature, we analysed 175 and 111 WHO Grade 4 glioma patients who received radiotherapy using CGGA RNA sequencing data and TCGA GBM data respectively. There are 26 WHO grade 4 IDH-mutant astrocytoma patients and 149 IDH-wildtype GBM patients in CGGA RNA sequencing data. Patients were divided into low-risk group and high-risk group based on the median risk score as the cut-off point (Fig. 6G, H). Most of the WHO grade 4 IDH-mutant astrocytoma patients were assigned to the low-risk group (Fig. 6I). Patients with IDH-wildtype GBM survived more in the low-risk group (Fig. 6J). Patients in the low-risk group had better prognosis than those in the high-risk group in IDH-wildtype GBM (P = 0.0233, Fig. 6K). Survival rate of patients in low-risk group is higher than that in high-risk group in WHO grade 4 IDH-mutant astrocytoma (P = 0.0924, Fig. 6L). As the number of patients is still very small, this result needs to be further verified by more cases. We also divided glioma patients of TCGA database into high- and low-risk groups according to the 4-gene prognostic signature (Fig. 6O–Q). Due to the fact that only seven patients in the TCGA database were WHO grade 4 IDH-mutant astrocytoma, survival analysis was not possible. Therefore, we conducted an overall analysis of WHO Grade 4 glioma patients. Result showed that patients in the low-risk group also had better prognosis than high-risk group in WHO Grade 4 gliomas (P = 0.025, Fig. 6R).

Most patients with WHO Grade 4 gliomas will undergo radiotherapy and chemotherapy according to the Stupp protocol^14^. Therefore, in order to remove the influence of confounding factors such as chemotherapy, patients in the radiotherapy group and the non-radiotherapy group were selected from the CGGA and TCGA databases. We analysed survival in the high- and low-risk groups of patients receiving radiotherapy alone and those not receiving radiotherapy. In TCGA database, there were 20 patients in radiotherapy group and 21 patients in non-radiotherapy group. In CGGA database, there were 24 patients in radiotherapy group and 30 patients in non-radiotherapy group. Results showed that high- and low-risk groups of patients receiving radiotherapy alone in TCGA have significant survival differences (P = 0.016, Fig. 6S), while patients who did not receive radiotherapy have no survival difference (P = 0.62, Fig. 6T). In CGGA database, although there was no statistically significant difference in survival among patients who receiving radiotherapy alone (P = 0.28, Fig. 6M), patients in the high-risk group showed a trend towards poorer prognosis compared to those in the low-risk group. For non-radiotherapy group patients, there was no statistically significant difference in survival between the high- and low-risk groups (P = 0.16, Fig. 6N). Moreover, patients in the low-risk group tend to have poorer prognosis. These results suggest that our 4-gene signature can predict radiotherapy efficiency in GBM patients.

## Discussion

IDH1 mutation has been identified as a prognostic marker that plays a major role in the tumourigenesis and treatment response of glioma^7^. IDH1 mutation is related to the carcinogenic progression of glioma, and its existence indicates a better clinical outcome regardless of the grade^15^. Several studies have confirmed that glioma cells with IDH1 mutations display increased sensitivity to radiation in vitro. Jacqueline Kessler et al. showed that overexpression of IDH1^R132^^H^ in glioma cells increases radiosensitivity, decreases cell proliferation slightly, and notably reduces cell migration^16^. Another study also found that glioma cells expressing IDH1^R132H^ mutant protein have reduced clonogenic ability after radiation compared with IDH1_wt_, hypothesizing that IDH1^R132H^ epigenetically reduces expression of TIGAR, a key controller of intracellular redox homeostasis, and that cells cannot eliminate IR-induced ROS^17^. Remco J. Molenaar et al. demonstrated that an IDH1^R132H^ small molecule inhibitor being developed for cancer treatment, AGI-5198, may reduce radiotherapy efficacy in glioma patients with IDH1^R132H18^. The researchers treated three glioma cell lines with AGI-5198 and radiation and found that the AGI-5198 reversed the DNA double-strand breaks and cell death caused by radiation^18^. Due to the importance of the IDH gene for glioma formation and prognosis, the 2021 WHO Classification of Tumors of the Central Nervous System classifies WHO grade 4 gliomas into WHO grade 4 IDH-mutant astrocytoma and IDH-wildtype GBM^4^.

These preclinical studies only explored whether IDH1 mutation is associated with cellular radiosensitivity, and there is currently little research on the relationship between IDH1 gene status and patient radiation therapy efficacy. We analysed the cellular phenotype and radiobiological consequences of overexpressing the IDH1^R132H^ mutant and IDH1_wt_ protein in human malignant glioma cells (U87MG and U251MG). IDH1 mutations effectively enhanced accumulation of radiation-induced γH2AX foci in these malignant glioma cells. Existing research shows that quantitation of γH2AX focus formation is closely related to DNA damage and repair^19^. In our study, glioma cells with the IDH1^R132^^H^ mutation showed the largest number of γH2AX foci at 0.5 h, 2 h, 6 h and 24 h after 5 Gy irradiation; the radiosensitivity of the cells expressing the mutant protein was increased and the DNA damage repair capacity deteriorated. In addition, we demonstrated using multiple assays that IDH1^R132H^ causes a significant reduction in the proliferation, migration and invasiveness of glioma cells. Therefore, at the cellular level, we proved that IDH1^R132H^ mutation can increase the radiosensitivity and apoptosis in glioma cells while reducing proliferation and invasion.

Through statistics of the glioma database and patients in our center, we confirmed that IDH1 gene status is indeed related to the efficacy of radiotherapy in glioma patients. We performed survival analysis on WHO grade 4 glioma patients who had undergone radiotherapy, and the results showed that WHO grade 4 IDH-mutant astrocytoma in both CGGA and TCGA had longer OS and PFS than IDH-wildtype GBM patients. We assessed the survival of 83 patients who received radiotherapy with known IDH1 gene status in our center, and the results showed better OS and PFS for WHO grade 4 IDH-mutant astrocytoma. The analysis results in the CGGA and TCGA databases are consistent with the results of our center, that is, among WHO grade 4 glioma patients receiving radiotherapy, IDH-wildtype GBM patients have a worse prognosis. Furthermore, a stratified analysis of patients in the CGGA database was conducted, and results showed that among patients receiving radiotherapy alone, WHO grade 4 IDH-mutant astrocytoma had better OS. While among patients who did not receive radiotherapy, there was no survival difference between WHO grade 4 IDH-mutant astrocytoma and IDH-wildtype GBM. However, due to the limited number of patients in stratified analysis, our results may be biased. A larger sample size analysis is needed to confirm this result. The above results suggest that there is a correlation between IDH gene status and radiosensitivity to some extent.

Based on differences in the radiotherapy effect and prognosis between WHO grade 4 IDH-mutant astrocytoma and IDH-wildtype GBM, we also analysed differences in gene expression between them. We screened WHO grade 4 glioma patients who received radiotherapy with known IDH1 gene status in the CGGA RNA array database. Through differential analysis and univariate and multivariate Cox regression survival analyses, we established a 4-gene signature (ADD3, GRHPR, RHBDL1 and SLC9A9) that was closely related to survival prognosis. Adducin 3 (ADD3) is a key assembly factor of the actin cytoskeleton found to be abnormally expressed in various cancers. It is downregulated in high-grade gliomas compared to low-grade gliomas, and its expression correlates with glioma stemness properties^20^. ADD3 plays a tumour inhibitory role in regulating glioma growth and angiogenesis^20,21^. Solute carrier family 9 member A9 (SLC9A9) encodes the endosomal Na+/H+ exchanger NHE9, a potential driver of glioma progression^22^. Kondapalli KC et al. showed that NHE9 promotes tumourigenesis, invasion, migration and radio/chemoresistance of GBM cells in vivo^23^. In GBM cells, silencing or inhibiting NHE9 reduces the formation of tumourspheres and improves the efficacy of EGFR inhibitors^23^. Rhomboid-like 1 (RHBDL1) is a proteolytic enzyme embedded within the plasma membrane that is significantly highly expressed in brain tissue. Glyoxylate reductase/hydroxypyruvate reductase (GRHPR) is a key enzyme of the glyoxylic acid cycle, and its deficiency can lead to primary hyperoxaluria type 2^24^. There is no relevant research about GRHPR and glioma.

Through literature search, we found that most of the current risk models for WHO grade 4 glioma patients are prognosis-related risk models. There are few risk models that can predict the efficacy of radiotherapy in WHO grade 4 gliomas. Du Z et al. established a radiosensitivity-related risk score model for lower-grade glioma (LGG) by analyzing hypoxia genes in the TCGA database^25^. Similarly, another study established a risk-score model related to radiation sensitivity by identifying DEGs between patients receiving radiation therapy and non-radiation therapy in LGG^26^. However, due to the high malignancy of WHO grade 4 gliomas, most patients will receive chemoradiotherapy according to the Stupp protocol. Only a few patients may receive radiotherapy alone or chemotherapy alone due to factors such as age or physical condition. Therefore, it is very difficult to establish a radiotherapy-related prognostic risk model in WHO grade 4 gliomas. Through our analysis and verification, the 4-gene signature can be used as an independent prognostic risk factor for the efficacy of radiotherapy in WHO grade 4 gliomas. In order to eliminate the influence of confounding factors such as chemotherapy, we conducted validation on patients who received radiotherapy alone and those who did not receive radiotherapy, respectively. Among TCGA patients, there was a significant survival difference between the high- and low-risk groups receiving radiotherapy alone, while there was no survival difference in patients who did not receive radiotherapy. This further indicates that the 4-gene risk-model has better predictive value in TCGA patients. Among CGGA patients, although there was no statistically significant difference in survival among patients receiving radiotherapy alone, the high-risk group had a trend towards poorer prognosis compared to the low-risk group. For patients who did not receive radiation therapy, there was no difference in survival between the high- and low-risk groups, and even showed poor prognosis in the low-risk group. The above results indicate that the 4-gene risk-model not only has a predictive effect on radiotherapy efficacy in the CGGA database, but also applies to patients in the TCGA database. This is also the advantage of the risk-model, which may have a wider applicability. After stratified analysis in CGGA patients, we find that the 4-gene signature is more suitable for patients with IDH-wildtype GBM. For WHO grade 4 IDH-mutant astrocytoma patients, there was no significant difference in survival between the high- and low-risk groups. However, from the Kaplan–Meier survival curve, the survival of patients in the high-risk group was even worse. Larger sample size analysis is needed. Because there are only seven WHO grade 4 IDH-mutant astrocytoma patients in TCGA database, survival analysis cannot be conducted. Therefore, we analyzed all WHO grade 4 gliomas. The Kaplan–Meier survival analysis results suggest that the high-risk group has poorer survival compared to the low-risk group. This also indicates that the risk model can be applied to TCGA patients. These results suggest that our 4-gene signature can be used as a stratification factor to predict prognosis after radiotherapy in WHO grade 4 gliomas especially IDH-wildtype GBM patients, though further research involving prospective clinical studies is needed.

## Conclusions

In conclusion, we provide compelling evidence that IDH1 mutation is related to the efficacy of radiotherapy in WHO grade 4 glioma patients. Experiments in vitro showed that overexpression of IDH1 mutant protein in GBM cells reduces radioresistance presenting a less malignant phenotype compared with IDH1wild-type. Furthermore, we established a 4-gene signature risk score model based on IDH1 gene status. This model can be used to predict response to radiotherapy and the prognosis of WHO grade 4 glioma patients.

### Supplementary Information

Supplementary Table S1.

Supplementary Table S2.

Supplementary Information.

## Data Availability

The datasets generated and/or analysed during the current study are available in the TCGA, CGGA and GEO database. https://portal.gdc.cancer.gov/projects/TCGA-GBM; http://www.cgga.org.cn/; https://www.ncbi.nlm.nih.gov/geo/query/acc.cgi?acc=GSE121720.
