# Supplementary material for: IDH1R132H mutation increases radiotherapy efficacy and a 4-gene radiotherapy-related signature of WHO grade 4 gliomas
Source: Sci Rep. 2023 Nov 11;13:19659. doi: 10.1038/s41598-023-46335-1 (PMC10640646; doi:10.1038/s41598-023-46335-1)
Supplement: Supplementary file 1 — Supplementary Table S1. [file 41598_2023_46335_MOESM1_ESM.docx]

| **Primer** | | **Forward (5’-3’)** |  | **Reverse (5’-3’)** |
| --- | --- | --- | --- | --- |
| GAPDH | CATGAGAAGTATGACAACAGCCT | |  | AGTCCTTCCACGATACCAAAGT |
| ADD3 | ACCAGCTCCTCCTAACCCA | |  | CATCCTTGCCATTTACTACC |
| GRHPR | CCAAGGAGCTAGAGCGAGGT | |  | GGTGGTATCTGTCAGGACATCTG |
| SLC9A9 | CCATGTTGACTTGGCTTCAG | |  | GAGGGGTCCTCCTTCAGATT |
| RHBDL1 | AGACCTACCACCCCGAGTACC | |  | GTTCCAGAAGACGGCGAAGA |

**Supplementary Table S1 Primer sequence for quantitative PCR**
