# Supplementary material for: IDH1R132H mutation increases radiotherapy efficacy and a 4-gene radiotherapy-related signature of WHO grade 4 gliomas
Source: Sci Rep. 2023 Nov 11;13:19659. doi: 10.1038/s41598-023-46335-1 (PMC10640646; doi:10.1038/s41598-023-46335-1)
Supplement: Supplementary file 2 — Supplementary Table S2. [file 41598_2023_46335_MOESM2_ESM.docx]

Supplementary Table S2: Uni-cox results of DEGs between WHO grade 4 IDH-mutant astrocytoma and IDH-wildtype GBM.

| **Gene** | **HR** | **HR.95L** | **HR.95H** | **P-value** |
| --- | --- | --- | --- | --- |
| ADD3 | 0.786055 | 0.646185 | 0.956199 | 0.016041 |
| ARHGAP22 | 0.762384 | 0.607453 | 0.95683 | 0.019248 |
| COX4I1 | 0.637855 | 0.429161 | 0.948034 | 0.026155 |
| GRHPR | 0.665525 | 0.490525 | 0.902957 | 0.008904 |
| KLF13 | 0.62876 | 0.436017 | 0.906707 | 0.01298 |
| LRRC8B | 0.740373 | 0.55441 | 0.988713 | 0.041662 |
| RBM17 | 0.698721 | 0.508065 | 0.960922 | 0.027443 |
| RHBDL1 | 0.688935 | 0.496727 | 0.955518 | 0.025575 |
| SCAMP2 | 0.610771 | 0.431081 | 0.865363 | 0.005547 |
| SLC9A9 | 0.57906 | 0.413143 | 0.81161 | 0.001515 |
| ZCCHC24 | 0.809941 | 0.66309 | 0.989313 | 0.038901 |
