# Supplementary figures and images for: IDH1R132H mutation increases radiotherapy efficacy and a 4-gene radiotherapy-related signature of WHO grade 4 gliomas
Source: Sci Rep. 2023 Nov 11;13:19659. doi: 10.1038/s41598-023-46335-1 (PMC10640646; doi:10.1038/s41598-023-46335-1)

**
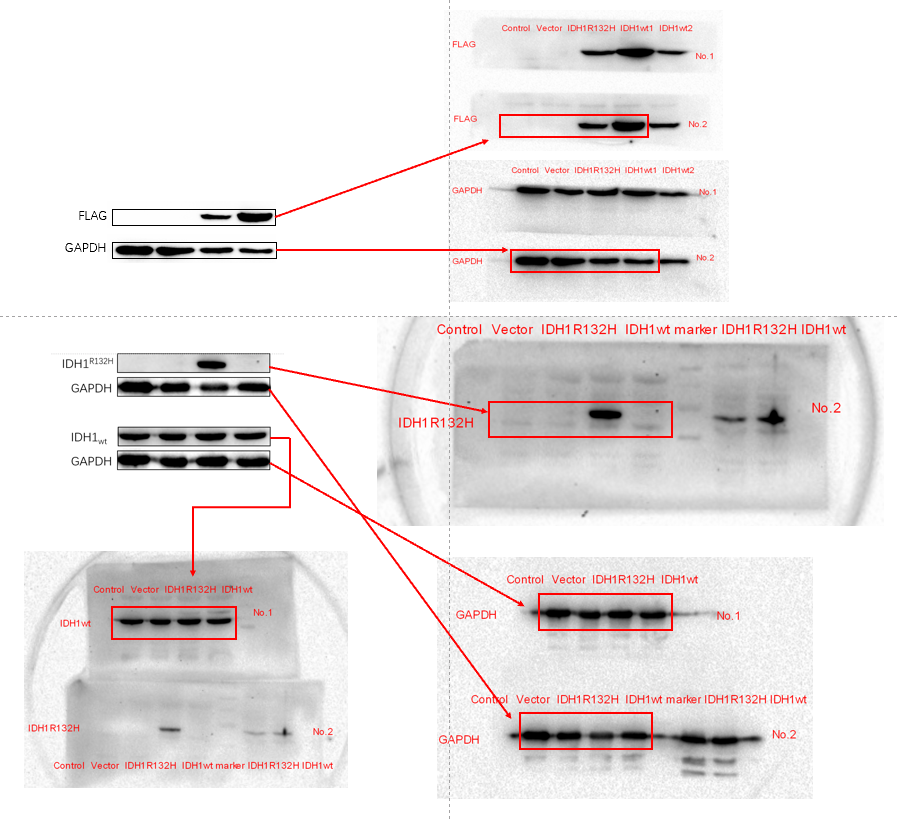
1.U87MG**

**
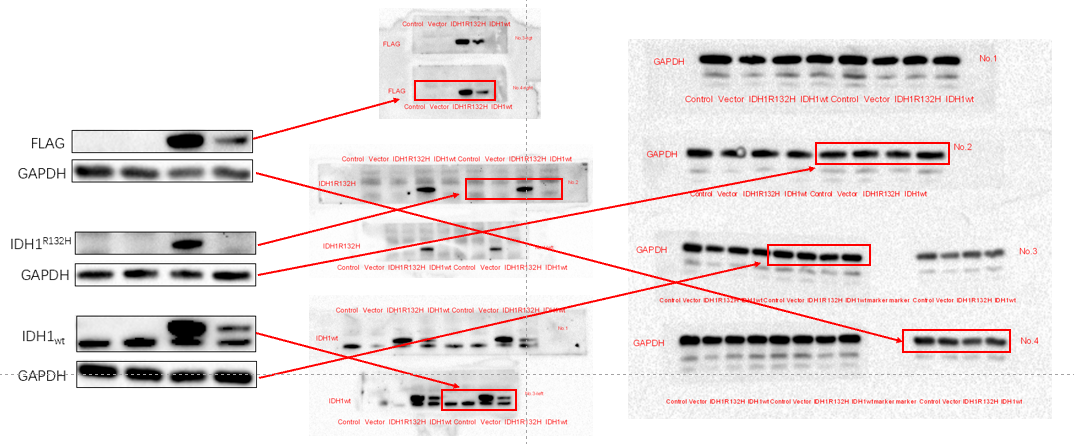
2.U251MG**

Supplement: Supplementary file 3 — Supplementary Information. [file 41598_2023_46335_MOESM3_ESM.docx]
